# Selenium status and its determinants in very old adults: insights from the Newcastle 85+ Study

**DOI:** 10.1017/S0007114523002398

**Published:** 2024-03-14

**Authors:** Giorgia Perri, John C. Mathers, Carmen Martin-Ruiz, Craig Parker, Jennifer S. Walsh, Richard Eastell, Kamil Demircan, Thilo S. Chillon, Lutz Schomburg, Louise Robinson, Tom R. Hill

**Affiliations:** 1 Human Nutrition and Exercise Research Centre, Centre for Healthier Lives, Population Health Sciences Institute, Faculty of Medical Sciences, Newcastle University, Newcastle upon Tyne NE2 4HH, UK; 2 MRC-Versus Arthritis Centre for Integrated Research into Musculoskeletal Ageing (CIMA), Faculty of Medical Sciences, Newcastle University, Newcastle upon Tyne NE2 4HH, UK; 3 BioScreening Core Facility, Campus for Ageing and Vitality, Newcastle University, Newcastle upon Tyne NE4 5PL, UK; 4 Department of Oncology and Metabolism, University of Sheffield, Sheffield S5 7AU, UK; 5 Institute for Experimental Endocrinology, Charité-Universitätsmedizin Berlin, Berlin 10115, Germany

**Keywords:** Selenium status, Very old adults, Biomarkers, Determinants

## Abstract

There is a dearth of data on Se status in very old adults. The aims of this study were to assess Se status and its determinants in 85-year-olds living in the Northeast of England by measuring serum Se and selenoprotein P (SELENOP) concentrations and glutathione peroxidase 3 (GPx3) activity. A secondary aim was to examine the interrelationships between each of the biomarkers. In total, 757 participants (463 women, 293 men) from the Newcastle 85+ Study were included. Biomarker concentrations were compared with selected cut-offs (serum Se: suboptimal 70 µg/l and deficient 45 µg/l; SELENOP: suboptimal 4·5 mg/l and deficient 2·6 mg/l). Determinants were assessed using linear regressions, and interrelationships were assessed using restricted cubic splines. Median (inter-quartile range) concentrations of serum Se, SELENOP and of GPx3 activity were 53·6 (23·6) µg/l, 2·9 (1·9) mg/l and 142·1 (50·7) U/l, respectively. Eighty-two percentage and 83 % of participants had suboptimal serum Se (< 70 µg/l) and SELENOP (< 4·5 mg/l), and 31 % and 40 % of participants had deficient serum Se (< 45 µg/l) and SELENOP (< 2·6 mg/l), respectively. Protein intake was a significant determinant of Se status. Additional determinants of serum Se were sex, waist:hip ratio, self-rated health and disease, while sex, BMI and physical activity were determinants of GPx3 activity. There was a linear association between serum Se and SELENOP, and nonlinear associations between serum Se and GPx3 activity and between SELENOP and GPx3 activity. These findings indicate that most participants had suboptimal Se status to saturate circulating SELENOP.

Se is an essential micronutrient for health. Se is incorporated as selenocysteine into selenoproteins, which are encoded by twenty-five separate genes containing an in-frame UGA codon. Selenoproteins are involved in neutralising reactive oxygen species and inflammation, both of which increase during ageing^(1,2)^. Excessive levels of reactive oxygen species are detrimental, ultimately leading to cellular senescence that accumulates over time and can contribute to the ageing phenotype^(2)^. A suboptimal Se status may contribute to disease risk and progression since Se status has been associated with inflammation^(3)^ and disease^(4)^ including metabolic dysfunction, CVD, cognitive decline, cancer and inflammatory bowel disease often following a U-shaped curve. Thus, there is an increasing interest in Se status with its ability to provide antioxidant properties through selenoproteins^(5)^. Therefore, focusing on dietary intakes and Se status of very old adults is important to help maintain and improve the ageing process. The richest dietary sources of Se are Brazil nuts, followed by protein-rich animal products, such as meat, offal and seafood, with lesser amounts in cereal products, fruits and vegetables^(6)^ with important regional differences due to differences in soil Se^(7)^. Mean dietary Se intake in older adults (≥ 75 years) who participated in the UK NDNS survey (*n* 167) (2008/09 and 2009/10) was 40 µg/d (42 µg/d for males and 39 µg/d for females)^(8)^. These values are well below the UK reference nutrient intake (75 and 60 µg/d, respectively, for men and women)^(9)^ and the US RDA (55 µg/d)^(10)^. Furthermore, 30 % of males and 52 % of females (*n* 224) aged ≥ 65 years had intakes below the lower reference nutrient intake of 40 µg/d^(9)^. To date, there is limited information on dietary intake of Se among very old adults. However, an earlier analysis using data from ≥ 85-year-olds (*n* 791) from the Northeast of England who participated in the Newcastle 85+ Study found that over 50 % had dietary Se intakes below the lower reference nutrient intake^(11)^.

Objective biomarkers of Se status have the potential to overcome some of the well-known limitations associated with dietary intake data. These biomarkers include the plasma selenoprotein extracellular glutathione peroxidase (GPx3), which plateaus at serum Se concentrations ranging from 70 to 100 µg/l^(6,10,12)^, and selenoprotein P (SELENOP), which plateaus at serum Se concentrations ranging from 90 to 125 µg/l^(13–17)^. Due to their sensitivity to low to moderate Se status, these plasma/serum biomarkers may be suitable for assessing Se status in older populations with lower Se intakes due to their sensitivity to low to moderate Se status^(18)^.

Generally with age, there is a reduction in energetic intake due to various physiological changes including appetite hormones, gut physiology, transit time, decreased energetic input and medication side effects. Thus, there is a reduction in nutrient intake and status in the body^(19)^. However, few studies have explored Se status in very old adults (Appendix Table 1). One of the few studies looking at Se status over time, a 9-year longitudinal study in France, found that 65-year-olds who survived the duration of the follow-up (*n* 1288) had a higher baseline serum Se (1·10 µmol/l; 86·5 µg/l) compared with those who died (*n* 101) (1·01 µmol/l; 79·5 µg/l)^(20)^. Furthermore, a study in free-living Italian adults (> 65 years) found mean Se concentrations of 0·94 µmol/l (74·0 µg/l), while institutionalised adults of > 85 years had lower concentrations of 0·8 µmol/l (62·9 µg/l)^(21)^.

In the UK, the NDNS survey reported that the median plasma Se concentration was 74·1 µg/l in free-living adults above 65 years (*n* 883). A review of Se status in very old adults found Se concentrations ranging from 88·9 µg/l in Italy (*n* 76, 86 years) to 113·7 µg/l in China (*n* 380, > 90 years)^(5)^. Furthermore, studies that inform the derivation of dietary Se recommendations rarely include data on very old adults (≥ 85 years). For example, the WHO/FAO of the United Nations recommendations are for adults up to 65 years^(22)^, the Scientific Committee for Food recommended a population reference intake for adults without consideration of older adults^(10)^ and the European Food Safety Authority pointed out that estimates of Se requirements to optimise selenoprotein concentrations come from studies involving adults aged 18 up to 64 years^(23)^. This evidence gap is important because very old adults are the fastest growing sub-population of older adults. In addition, there is great heterogeneity in this sub-group ranging from those who are relatively healthy to the larger proportion who suffer from multi-morbidity^(24)^. These differences can lead to difficulties in determining dietary requirements and in preparing public health recommendations. The aims of this study were: (1) to assess the Se status (by measuring total serum Se and SELENOP concentrations and GPx3 activity) and its determinants in 85-year-old adults from the Newcastle 85+ Study and (2) to quantify relationships between serum Se concentration and SELENOP concentration and GPx3 activity.

## Methods

### Study population

This study used participant data and samples collected at baseline in the Newcastle 85+ Study, which is a longitudinal study of health outcomes and trajectories of 1042 participants born in 1921. This study explores the cross-sectional data of these participants. Participants were registered with GPs from North Tyneside or Newcastle upon Tyne primary care trusts and were recruited from sixty-four centres (Northeast England). The only exclusions were individuals with end-stage terminal illness and those who could not be visited by a lone nurse without posing risks. Baseline assessments used in these cross-sectional analyses were undertaken between 2006 and 2007^(24)^.

### Ethics approval

The study was conducted in accordance with the Declaration of Helsinki. The Newcastle and North Tyneside local research ethics committee (06/Q0905/2) approved the research and all participants provided written and informed consent. For those who lacked capacity, a carer or relative provided consent in line with the UK Mental Capacity Act 2005.

### Socio-economic, lifestyle and other covariates

Baseline assessments (2006/2007) were undertaken in each participant’s place of residence (home or an institution) by research nurses who underwent 6 weeks of training^(24)^. Questionnaires, functional tests, fasting blood samples, medical record reviews, dietary intakes and body weight measurements were taken at the initial health assessment (online Supplementary Material Table 1)^(25,26)^. General practice medical records were analysed to obtain information on current medication, service usage and disease information.

Participants were classified into the National Statistics Socio-Economic Classification three-class scheme based on their main previous occupation^(27)^. Education was determined by the duration of full-time higher education. Self-rated health was subjectively assessed and categorised as excellent/very good, good or fair/poor. Cognitive impairment was classified as scores ≤ 25 points out of 30, on the Standardised Mini-Mental State Examination. BMI was calculated as kg weight/m^2^ height and fat-free mass (kg) was estimated using the Tanita-305 body fat bioimpedance instrument (Tanita Corp.). Medication use, including non-prescribed medication, was determined using GP records and packaging at participant interviews. Smoking and alcohol questionnaires recorded habit status, frequency, past habits and duration. Physical activity was assessed using a purpose-built questionnaire and validated by comparison with accelerometery^(28)^. Se intake (µg/d), total energy intake (kcal) and protein intake (g) were determined using the 24-h multiple pass recall^(11,29)^. Disease count was calculated using a selected list of chronic diseases (online Supplementary Material Table 2)^(24)^.

### Biomarkers of selenium status

Few studies have used blood samples stored for as long as 16 years for the estimation of Se status, but a review of the literature suggested that Se in serum is stable over long time periods (at least 10–15 years) and that serum SELENOP is stable during freeze-thaw cycles over long periods of time^(30)^. Serum samples stored for > 10 years retained linear correlations between the biomarkers of Se status suggesting stability^(31–34)^. Consequently, baseline blood samples collected in 2006/2007 (*n* 757) that had been stored at –80°C were analysed for biomarkers of Se status by scientists blinded to the data. This included serum Se (µg/l) and SELENOP (mg/l) concentrations and GPx3 activity (U/l). Total serum Se concentration was measured by the total reflection X-ray fluorescence using a bench-top spectrometer (S4 T-STAR, Bruker Nano GmbH) for 2000 s per sample. As a reference, a gallium standard (1000 µg/l) was used to dilute participant serum in a 1:2 ratio, as described elsewhere^(34)^. Eight microlitres of the diluted solution was applied to polished quartz glass slides (Bruker Nano GmbH), and these were dried overnight in an incubator at 37°C. Serum standard Seronorm was used as a control (concentration of 87 µg/l determined by ICP-MS) (Seronorm™ Trace Elements Serum L-1 SeronormTM, Cat#201405, Lot-Nr 1309438, Sero AS). The inter- and intra-assay CV from the ten assay runs were below 10 % at 76–99 µg/l. The total reflection X-ray fluorescence method is comparable to inductively coupled plasma optical emission spectroscopy and ICP-MS and produces congruent results^(35,36)^. Serum SELENOP concentration was analysed using a validated immunoluminometric, commercial ELISA (selenOtest, selenOmed GmbH). The sandwich ELISA used 5 µl of serum and human SELENOP-specific monoclonal antibodies in addition to three controls that represented the assay’s working range. Absorbance at 450 nm was measured using a photometer, standards of known concentration were included in each assay run and a standard curve was fitted to the data. Each sample was measured in duplicate, and the mean SELENOP concentrations were calculated. The high, low and medium SELENOP concentration standards produced by the manufacturer for the selenOtest were each measured in twelve assays (i.e. 96-well plates) and yielded results well within the specified range defined by selenOmed, with CV between runs of 7·3 % (high standard), 12·4 % (low standard) and 3·7 % (middle standard). The concentrations measured were within the specified range of the standard (provided by selenOmed GmbH), and the three controls (low, medium, high) had SELENOP concentrations of 0·58, 2·3 and 9·3 mg/l, as indicated by the manufacturer. GPx3 activity was analysed using a coupled-enzyme reaction measuring NADPH consumption^(37)^. Serum samples (including control serum) were incubated at 20°C with 0·27 mg/ml NADPH, 1 mM sodium azide (NaN3), an enzyme buffer containing 3·4 mM reduced glutathione and 0·3 U/ml glutathione reductase. The reaction was initiated using hydrogen peroxide. At 340 nm, reductions in UV absorption were proportional to NADPH consumption, which reflected GPx3 activity. Assays were carried out in triplicate and the mean activity was reported. The inter-assay CV was below 15 % and intra-assay CV was below 10 % as noted in these analyses and another study^(38)^.

### Cut-offs for selenium status

Currently, there is no consensus for suboptimal selenoprotein concentrations or activity for very old adults; therefore, cut-offs were selected based on average values or centiles in other populations^(39)^ (Appendix Table 2). For serum Se, a lower cut-off of 70 µg/l from a multicounty analysis was used since this population of very old adults had a suboptimal serum Se concentration^(6)^ (Appendix Table 2). The SELENOP cut-off (4·5 mg/l) was determined as the mean SELENOP concentrations devised from the EPIC-Europe cohort^(34)^ (mean 3·9 and 4·3 mg/l in males and females) and an American Se supplementation study^(15)^ (mean 5·5 mg/l). Additionally, cut-offs for deficiency were used to allow comparisons between those participants with suboptimal and deficient Se status. Both the deficient serum Se cut-off (< 45 µg/l) and SELENOP (< 2·6 mg/l) were devised using the 2·5th centile from a healthy sub-group (*n* 1915, 51 % females, 59 (sd 7) years at blood collection) of the EPIC cohort study^(40)^.

### Statistical analyses

IBM statistical software package version 27.0 (SPSS) was used to perform the exploratory and statistical analyses, and *P* < 0·05 was considered statistically significant. To determine normality of the continuous variables, the Shapiro–Wilk test and quantile–quantile (QQ) plots were used. Se status biomarkers were used as continuous variables in the main analyses and categorised into optimal and suboptimal and non-deficient and deficient sub-groups based on biologically relevant cut-offs as described above. Descriptive statistics were used to summarise the baseline characteristics of all participants and of those with biomarker concentrations above and below the selected cut-offs. Differences in characteristics between the cut-offs were assessed using *χ* ^2^ test (categorical) and Kruskal–Wallis (for ordered and non-normally distributed data). R Studio was used with the libraries ggplot, ggvenn and venndiagram to plot a Venn diagram indicating the participants who had suboptimal and deficient concentrations of serum Se and SELENOP using the same cut-offs described above. R studio was also used with the libraries ggplot, rms and hmisc to produce restricted cubic splines to determine nonlinearity using likelihood ratio test. A *P*_non-linearity_ < 0·05 was considered as a relationship deviating from linearity. Ordinary least squares regression models with three knots at the 10th, 50th and 90th percentiles were visualised as plots.

### Predictors of baseline selenium status

Linear regression models were used to determine the predictors of each of the biomarkers of Se status (serum Se, SELENOP and GPx3 activity). The Se biomarkers were the dependent variables, and the independent variables were sex (men/women, binary); occupational status (routine/manual, intermediate, managerial/professional occupations, categorical); education (0–9, 10–11, ≥ 12 years, categorical); self-rated health (excellent/very good, good, fair/poor, ordinal); energy intake (continuous); protein intake (continuous); Se intake (continuous); medication use (continuous); BMI (continuous); fat-free mass (continuous); waist:hip ratio (continuous); Standardised Mini-Mental State Examination (continuous); disease count (0–1, 2, ≥ 3, categorical); number of medications (continuous); smoking status (current, former, never, categorical); physical activity (continuous); alcohol drinker (yes/no binary) and high-sensitivity C-reactive protein (continuous). The chosen covariates were based on findings from previous literature^(18)^.

The analyses were repeated using logistic regression models to determine the predictors of each biomarker of Se status when categorised as a binary variable using the selected cut-offs (70 and 45 µg/l for serum Se; 4·5 and 2·6 mg/l for SELENOP).

## Results

### Baseline selenium status and participant characteristics

Table 1 summarises the 5th–95th percentiles of biomarkers of Se status. The median concentrations were serum Se 53·6 µg/l, SELENOP 2·9 mg/l, GPx3 activity 142·1 U/L and Se intake 39·1 µg/d. Baseline characteristics of all participants and for those with biomarker concentrations above and below the suboptimal and deficient cut-offs are shown in Table 2 and Appendix Table 3, respectively. The median Se intake for all participants did not differ significantly between participants with suboptimal or optimal Se concentrations (< 70 or ≥ 70 µg/l) (*P* = 0·06) but did for those with deficient and non-deficient Se concentrations (< 45 or ≥ 45 µg/l) (*P* < 0·001). Those with suboptimal Se concentration (< 70 µg/l) were more likely to be male (*P* = 0·010), live in institutions (*P* = 0·002), have higher physical activity (*P* = 0·001), higher medication usage (*P* < 0·001), lower cognitive score (*P* = 0·007), higher high-sensitivity C-reactive protein (*P* = 0·002), higher free thyroxine (T4) (*P* = 0·03), higher BMI (*P* = 0·002) and higher fat-free mass (*P* < 0·001). Those with suboptimal SELENOP concentration (< 4·5 mg/l) were more likely to have lower protein intake (*P* = 0·03) and higher waist:hip ratio (*P* = 0·02) and free T_4_ (*P* = 0·021). Those with deficient Se concentration (< 45 µg/l) were more likely to live in institutions (*P* < 0·001), have lower protein intake (*P* = 0·03), higher medication usage (*P* < 0·001), lower alcohol intake (*P* = 0·006), lower physical activity (*P* < 0·001), lower cognitive score (*P* < 0·001), higher high-sensitivity C-reactive protein (*P* = 0·005) and lower free tri-iodothyronine (T_3_) (*P* < 0·001). Those with deficient SELENOP concentration (< 2·6 mg/l) were more likely to have lower education (*P* = 0·04), lower protein intake (*P* = 0·005) and have higher free T_4_ (*P* < 0·001).

**Table 1.** Biomarkers of selenium status (serum Se, selenoprotein P, glutathione peroxidase 3 activity) of study participants represented by percentiles (5–95th)

Biomarker of Se status *n* 755	Percentiles						
	5	10	25	50	75	90	95
Serum Se (µg/l)	26·64	32·90	42·80	53·60	66·44	77·10	84·64
SELENOP (mg/l)	0·90	1·33	2·04	2·93	3·97	5·08	5·66
GPx3 activity (U/L)	63·78	79·56	107·4	142·10	179·30	210·42	231·96

SELENOP, selenoprotein P; GPx3, glutathione peroxidase.

**Table 2.** The characteristics of study participants represented by suboptimal Se status cut-offs (Numbers and percentages; mean values and standard deviations)

	All participants *n* 757		Se < 70 µg/l		Se ≥ 70 µg/l			SELENOP < 4·5 mg/l		SELENOP ≥ 4·5 mg/l		
Characteristics	*n*	%	*n*	%	*n*	%	*P*	*n*	%	*n*	%	*P*
Socio-demographic factors												
Women	61·1	461	79·0	364	21·0	97	0·010	80·9	373	19·1	88	0·07
Men	38·9	293	86·3	253	13·7	40		86·0	252	14·0	41	
Education *n* 743												
0–9 years	64·2	477	82·8	395	17·2	82	0·48	81·8	390	18·2	87	0·33
10–11 years	23·6	175	78·9	138	21·1	37		84·0	147	16·0	28	
12+ years	12·2	91	80·2	73	19·8	18		87·9	80	12·1	11	
Living in institution *n* 755												
Yes	8·9	67	95·5	64	4·5	3	0·002	88·1	59	11·9	8	0·24
No	91·1	688	80·5	554	19·5	134		82·4	567	17·6	121	
	M	sd	M	sd	M	sd		M	sd	M	sd	
Diet-related factors												
Total energy kcal *n* 732	1688·6	511·0	1688·6	524·1	1689·0	450·9	0·82	1679·4	508·8	1733·3	521·4	0·31
Protein intake g *n* 732	64·2	22·3	64·0	22·5	65·1	21·9	0·68	63·2	21·5	69·4	25·5	0·03
Se intake µg/d *n* 732	45·3	29·8	44·6	30·3	48·2	27·5	0·06	44·6	29·0	48·8	33·5	0·33
Lifestyle factors												
Number of medications *n* 732	6·3	3·8	6·6	3·8	5·3	3·7	< 0·001	6·2	3·8	6·8	4·0	0·21
	*n*	%	*n*	%	*n*	%		*n*	%	*n*	%	
Total medication *n* 753												
0–2	16·7	126	70·6	89	29·4	37	< 0·001	85·7	108	14·3	18	0·62
3–5	26·7	201	78·6	158	21·4	43		81·6	164	18·4	37	
≥ 6	56·6	426	86·6	369	13·4	57		82·9	353	17·1	73	
Alcohol drinker *n* 751												
Yes	62·3	468	82·1	384	17·9	84	0·88	83·8	392	16·2	76	0·38
No	37·7	283	81·6	231	18·4	52		81·3	230	18·7	53	
Physical activity score *n* 748												
Low	21·7	162	90·7	147	9·3	15	0·001	82·7	134	17·3	28	0·95
Medium	43·0	322	81·4	262	18·6	60		83·5	269	16·5	53	
High	35·3	264	76·9	203	23·1	61		82·6	218	17·4	46	
Health-related factors												
Self-rated health *n* 738												
Excellent/very good	40·9	302	77·8	235	22·2	67	0·07	81·1	245	18·9	57	0·55
Good	37·7	278	83·1	231	16·9	47		84·5	235	15·5	43	
Fair/poor	21·4	158	86·1	136	13·9	22		82·3	130	17·7	28	
	M	sd	M	sd	M	sd		M	sd	M	sd	
SMMSE *n* 753	26·1	4·9	25·9	5·2	27·2	3·4	0·007	26·1	4·9	26·3	5·2	0·35
hsCRP mg/l *n* 753	6·9	14·2	7·4	15·2	4·3	9·5	0·002	6·9	14·8	6·5	12·4	0·51
Free T_4_ pmol/l *n* 742	15·6	2·7	15·7	2·7	15·1	2·4	0·03	15·7	2·7	15·0	2·3	0·02
Free T_3_ pmol/l n - 743	4·5	0·5	4·5	0·5	4·6	0·5	0·06	4·5	0·5	4·5	0·5	0·55
Anthropometry												
BMI *n* 674	24·4	4·4	24·6	4·4	23·4	3·9	0·002	24·3	4·3	24·7	4·5	0·57
Fat-free mass *n* 689	45·2	9·0	45	9·0	42·9	8·6	< 0·001	45·4	9·1	44·1	8·7	0·16
Waist:hip ratio *n* 685	0·89	0·08	0·9	0·08	0·9	0·07	0·06	0·88	0·08	0·87	0·07	0·02

SELENOP, selenoprotein P; SMMSE, Standardised Mini-Mental State Examination; hsCRP, high-sensitivity C-reactive protein; Free T_4_, free thyroxine; Free T_3_, free triiodothyronine.

The characteristics are displayed for all participants and those with concentrations above and below cut-offs for suboptimal/optimal Se and selenoprotein status. *P* values determined using Mann Whitney U or Kruskal Wallis tests for continuous variables or *χ* ^2^ test for categorical variables. *P* values indicate the difference between suboptimal and optimal status.

Most participants (81·8 %, *n* 619) had suboptimal serum Se concentration, that is, below 70 µg/l, and suboptimal (82·8 %, *n* 627) SELENOP concentration, that is, below 4·5 mg/l. Participants with optimal Se status as judged by serum Se concentration ≥ 70 µg/l were more likely to be optimal for SELENOP (*P* < 0·001). Almost a third of participants (30·6 %, *n* 232) had deficient serum Se concentration, that is, below 45 µg/l, and over a third (39·9 %, *n* 302) had deficient SELENOP concentration, that is, below 2·6 mg/l.

### Relationships between biomarkers of selenium status at baseline

**Fig. 1.**
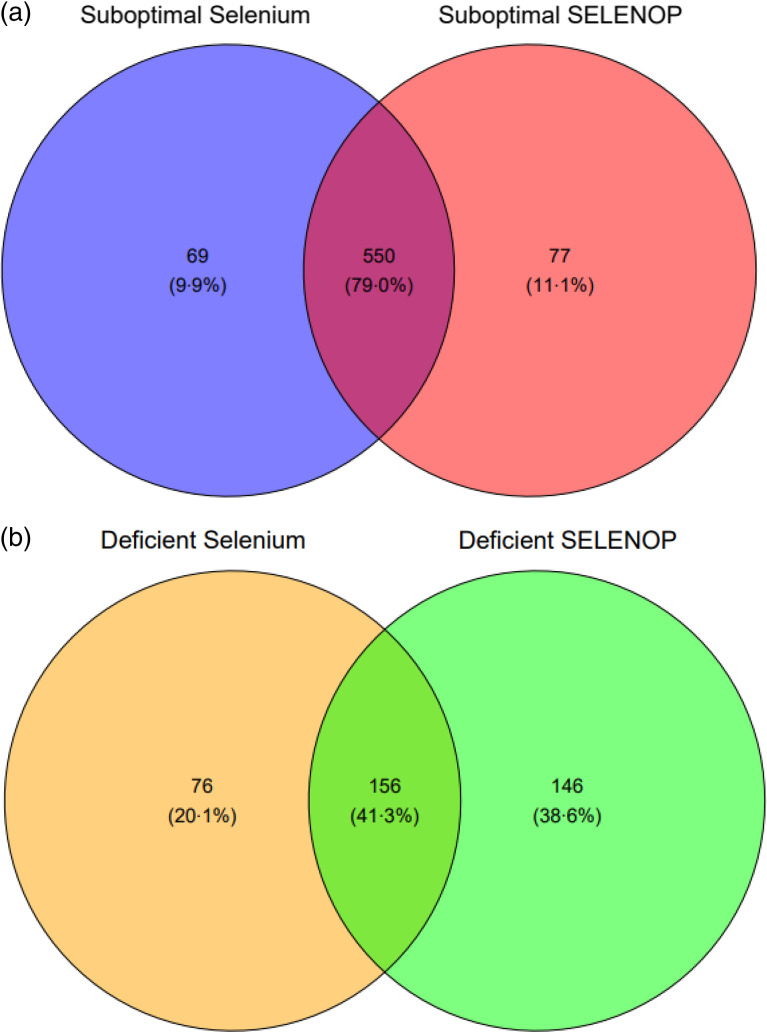
(a) Venn diagram depicting the overlap of suboptimal concentrations of biomarkers of Se status. Suboptimal concentrations were defined in those who had serum Se < 70 µg/l and selenoprotein *P* < 4·5 mg/l. (b) Venn diagram depicting the overlap of deficient concentrations of biomarkers of Se status. Deficient concentrations were defined as serum Se < 45 µg/l and selenoprotein *P* < 2·6 mg/l.

A Venn diagram (Fig. 1) revealed the overlap between the participants who were considered to have suboptimal Se status. There was a 79 % overlap in those who were considered suboptimal for serum Se (< 70 µg/l) and for SELENOP (< 4·5 mg/l). Considering Se deficiency, that is, serum Se concentrations below 45 µg/l and SELENOP below 2·6 mg/l (2·5th centile of EPIC)^(40)^, there was an overlap of 41 % of participants. Restricted cubic splines and likelihood ratio tests revealed a linear association between serum Se and SELENOP (*χ* ^2^ (df = 1) = 0·96, *P* = 0·33) and nonlinear associations between serum Se and GPx3 activity (*χ* ^2^ (df = 1) = 7·88, *P* = 0·005) and between SELENOP and GPx3 activity (*χ* ^2^ (df = 1) = 4·86^E+1^, *P* = 3·20^E-12^) (Appendix Fig. 1).

### Predictors of biomarkers of selenium status

In the fully adjusted regression models (Table 3), a higher protein intake was a significant predictor of each biomarker of Se status. In addition, serum Se concentration was higher in females (*β* 8·38 (sd 3·04), *P* = 0·006), those with higher waist:hip ratios (*β* 24·66 (sd 12·19), *P* = 0·04), lower in those with poor self-rated health (*β* –2·31 (sd 1·11), *P* = 0·04) and with a higher disease count (*β* –1·07 (sd 0·51), *P* = 0·04). Similarly, GPx3 activity was higher in females (*β* 30·92 (sd 8·23), *P* < 0·001) and lower in those with higher BMI (*β* –2·17 (sd 0·74), *P* = 0·003) and those who were more physically active (*β* –1·32 (sd 0·60), *P* = 0·03).

**Table 3.** Significant predictors of the biomarkers of Se status (Beta-coefficients and standard errors)

					Model
	Variable	*β*	se	95 % CI	*P*
Serum Se	Constant	45·60	14·35	17·41, 73·79	0·002
	Female	8·38	3·04	2·41, 14·36	0·006
	Waist:hip ratio	24·66	12·19	0·71, 48·61	0·04
	Protein intake (g)	0·13	0·05	0·02, 0·23	0·03
	Self-rated health	–2·31	1·10	–4·49, −0·14	0·04
	Disease count	–1·07	0·51	–2·06, −0·01	0·04
SELENOP	Constant	2·94	1·15	0·68, 5·21	0·01
	Protein intake (g)	0·01	0·004	0·01, 0·02	0·002
GPx3 activity	Constant	144·99	38·84	68·70, 221·28	< 0·001
	Female	30·92	8·23	14·75, 47·08	< 0·001
	BMI	–2·17	0·74	–3·62, −0·73	0·003
	Protein intake (g)	0·35	0·15	0·07, 0·64	0·02
	Physical activity	–1·32	0·60	–2·49, −0·14	0·03

SELENOP, selenoprotein P; GPx3, glutathione peroxidase.

When using the selected suboptimal cut-off, a lower medication use (3–4 medications compared with > 6) was a significant predictor of optimal Se concentration (≥ 70 µg/l) (Exp(*β*) 2·03 (1·045–3·937) *P* = 0·04) and non-deficient Se concentration (≥ 45 µg/l) (Exp(*β*) 1·68 (1·039–2·729) *P* = 0·03) (1–2 medications compared with > 6) (Exp(*β*) 2·10 (1·062–4·134) *P* = 0·03). A higher disease count was a significant predictor of deficient Se concentration (< 45 µg/l) (Exp(*β*) 0·85 (0·747–0·964) *P* = 0·01). A higher protein intake was a predictor of optimal SELENOP concentration (≥ 4·5 mg/l) (Exp(*β*) 1·02 (1·007–1·040) *P* = 0·005) and non-deficient SELENOP concentration (≥ 2·6 mg/l) (Exp(*β*) 1·02 (1·003–1·029) *P* = 0·02) and non-deficient Se concentration (≥ 45 µg/l) (Exp(*β*) 1·02 (1·001–1·031) *P* = 0·03).

## Discussion

Over 80 % of the 85-year-old adults in this study had suboptimal serum Se and SELENOP concentrations and over 40 % had deficient Se status when judged by reference values derived from healthy adults of all age categories. Higher protein intake was associated with higher concentrations of all three biomarkers of Se status. In addition, sex, waist:hip ratio, disease count and self-rated health predicted serum Se concentration and sex, BMI and physical activity predicted GPx3 activity while only protein intake predicted SELENOP concentration. The negative association of number of medications taken and suboptimal SELENOP concentrations, which is in line with recent research^(41)^, indicates that concurrent illness is a noteworthy risk factor for suboptimal Se status, particularly among very old adults.

Many studies have reported suboptimal Se intakes in older adults^(6,42,43)^, but measurements of multiple functional biomarkers (serum Se, SELENOP, GPx3 activity) of Se status are scarce. Serum Se and SELENOP concentrations in the 85-year-old adults in the present study are generally lower than in other UK older adults (generally for adults < 80 years) (Appendix Table 1)^(44–46)^. For example, in a British cohort (*n* 119) (50–64 years), mean SELENOP concentration was 4·9 mg/l^(14)^. Furthermore, in the EPIC-Europe cohort (n 966, mean 60 years, 70 % women from Denmark, France, Germany, Greece, Italy, the Netherlands, Norway, Spain, Sweden and UK), participants had a mean serum Se of 81·9 µg/l and SELENOP of 4·3 mg/l^(34)^. Likewise, in the Malmö Preventive Project (*n* 4366, mean 70 years), participants in quintiles 2–5 had SELENOP concentrations ranging from 4·3 to 20 mg/l^(33)^. A study of southeastern US adults (*n* 191, 40–79 years, 61 % earning below $15 000 per year) found a mean serum Se of 117·6 µg/l, a mean SELENOP concentration of 4·7 mg/l and a mean GPx3 activity of 132·0 U/L^(47)^. In that study, GPx3 activity was lower than the activity of the Newcastle 85+ Study participants despite the younger age range. This difference may be driven by socio-economic status. Furthermore, there is wide heterogeneity from the literature in defining ‘adequate’, ‘optimal’ and ‘deficient’ concentrations of Se biomarkers. Authoritative reports on dietary Se requirements often vary because of differences in techniques and criteria used including which Se biomarkers are chosen, reference cohort is analysed and the criteria for devising the respective cut-offs. For example, in the UK, dietary recommendations for Se established by the Committee on Medical Aspects of Food and Nutrition Policy (COMA) in 1991 were based on the Se intake required to maximise the activity of GPx3^(48)^. It was suggested that GPx3 activity plateaued when whole blood Se was 100 µg/l, that is, the system was saturated^(9,49)^. Alternatively, as used in our analyses, a lower cut-off of 70 µg/l has been suggested for the optimisation of GPx3 activity^(6,12)^ (Appendix Table 2). More recently, other organisations (D-A-CH and European Food Safety Authority (EFSA))^(13,23,50)^ have used the achievement of a plateau in SELENOP when considering the dose–response relationship between Se intake and SELENOP to indicate optimal Se intake.

The results are in agreement with former studies indicating that SELENOP and GPx3 activity require a higher Se intake for full and saturated expression of these two biomarkers^(13,14)^. This may be because both serum selenoproteins are towards the lower end of the selenoprotein hierarchy^(51–53)^, which may mean that higher intakes of Se are needed to maximise the expression. This interrelationship is further complicated by tissue hierarchy, where organs lower in the hierarchy such as the liver, where SELENOP is synthesised, are depleted first, prior to endocrine tissues or the central nervous system^(54,55)^. Likewise, the kidney, where GPx3 is synthesised, is also compromised in times of Se deficiency^(56)^.

The prevalent suboptimal (over 81 % of participants) and deficient (over 40 % of participants) Se status observed in this population of very old adults may indicate suboptimal Se intake. As expected, protein intake was a significant predictor of all three biomarkers of Se status. Protein-rich foods are generally rich in Se, and intakes of such foods have correlated with SELENOP in older women^(16)^. Waist:hip ratio predicted serum Se and BMI predicted GPx3 activity. Higher adiposity measures are associated with increased inflammation and greater oxidative stress^(57)^, which is negatively associated with Se status^(58–61)^. Furthermore, females had higher concentrations of all three biomarkers of Se. In contrast, males had higher serum Se concentrations in a Spanish study of ≥ 80-year-olds^(62)^, although sex was not associated with differences in Se concentrations in other studies^(18,63,64)^. Poor self-rated health and a higher disease count were associated with lower concentrations of serum Se and were also predictors of Se status in another British cohort of older adults^(18)^. Higher levels of physical activity were negatively associated with GPx3 activity. In contrast, in a 12-week walking programme, GPx3 activity increased in sedentary, postmenopausal women (65 years)^(65)^. Changes in GPx3 activity can be associated with oxidative stress, whereby GPx3 functions to detoxify free radicals that increase after intense physical activity^(66)^. However, in our population, higher physical activity was associated with lower GPx3 activity, which highlights the need for more research to explore the relationship between GPx3 activity and physical activity among very old adults.

Like other nutrients, there are potential health consequences to suboptimal Se status. Our analyses have found associations between lower Se status and higher medication count, poorer self-rated health, lower cognitive performance, higher inflammation (high-sensitivity C-reactive protein), higher free T_^4^_, lower free T_3_, higher BMI, fat-free mass and waist:hip ratio, although these are cross-sectional associations and do not imply causation. On the other hand, very old adults may have adapted to a lower Se supply and have mechanisms to cope with this limited intake without a major detriment to health^(67)^. In a study of Se-deficient rats, there was no negative effect on lifespan – indeed, lifespan was increased for those with restricted Se intake^(68)^. These findings may indicate a pro-longevity effect of suboptimal Se status. Although, to date, no studies in humans have found these associations and often reveal the converse, an increase in ageing and disease, thus pro-longevity mechanisms appear counterintuitive given the many reports of increased health risks with suboptimal Se status^(69)^. These findings stress the need for future longitudinal analyses to determine the potential relevance of suboptimal and deficient Se status in very old adults.

This is the first cross-sectional study to date that has measured Se status using three specific biomarkers in ≥ 85-year-old adults. A major strength to this study is the availability of three established biomarkers of Se status. Another key strength of this study is the large sample size (*n* 757) with inclusion of all participants regardless of living status. However, biomarkers of Se status were only assessed in baseline samples, and thus, other characteristics could not be followed up. Further research is needed to establish the cut-offs for optimal Se status in very old adults especially given the documented U-shaped relationship between Se status, chronic disease and mortality risk^(4,70)^.

### Conclusions

When judged by the criteria for optimal Se status derived using dietary reference intakes, there was a high prevalence of suboptimal Se status in this population of 85-year-olds. This may be due to the fact the Se dietary reference intakes are based on experimental studies using younger adults. The clinical consequences of these findings are currently unclear, so it will be important to undertake longitudinal studies of the relationship between Se intake and Se status at baseline and subsequent health outcomes. It is likely that those individuals with Se and SELENOP deficiencies (below 45 µg/l and 2·6 mg/l, respectively) are at particularly elevated health risks, as has been seen in studies with younger adults. However, this hypothesis will need to be tested using longitudinal observational studies.

## Supporting information

Perri et al. supplementary material 1 Perri et al. supplementary material

Perri et al. supplementary material 2 Perri et al. supplementary material

## Supplementary material

For supplementary materials referred to in this article, please visit https://doi.org/10.1017/S0007114523002398

## References

[1] Fairweather-Tait SJ , Bao Y , Broadley MR , et al. (2010) Selenium in human health and disease. Antioxid Redox Signaling 14, 1337–1383. 10.1089/ars.2010.3275 20812787

[2] Chrousos GP (2009) Stress and disorders of the stress system. Nat Rev Endocrinol 5, 374–381. 19488073 10.1038/nrendo.2009.106

[3] Tseng CK , Ho CT , Hsu HS , et al. (2013) Selenium is inversely associated with interleukin-6 in the elderly. J Nutr Health Aging 17, 280–284.23459983 10.1007/s12603-012-0376-6

[4] Sun Y , Wang Z , Gong P , et al. (2023) Review on the health-promoting effect of adequate selenium status. Front Nutr 10, 1136458. 37006921 10.3389/fnut.2023.1136458 PMC10060562

[5] Robberecht H , De Bruyne T , Davioud-Charvet E , et al. (2019) Selenium status in elderly people: longevity and age-related diseases. Curr Pharm 25, 1694–1706. 10.2174/1381612825666190701144709 31267854

[6] Combs GF Jr (2001) Selenium in global food systems. Br J Nutr 85, 517–547. 11348568 10.1079/bjn2000280

[7] Rayman MP (2008) Food-chain selenium and human health: emphasis on intake. Br J Nutr 100, 254–268. 18346308 10.1017/S0007114508939830

[8] Roberts C , Steer T , Maplethorpe N , et al. (2018) Results from Years 7 and 8 (Combined) of the Rolling Programme (2014/2015 to 2015/2016). *National Diet and Nutrition Survey (NDNS) Public Health England*. https://dera.ioe.ac.uk//id/eprint/31298 (accessed January 2023).

[9] Scientific Advisory Committee on Nutrition (SACN) (2013) SACN Position Statement on Selenium and Health. https://www.gov.uk/government/publications/sacn-statement-on-selenium-and-health-2013 (accessed April 2023).

[10] Institute of Medicine Panel (2000) *Dietary, Antioxidants Related, Compounds*. Dietary Reference Intakes for Vitamin C, Vitamin E, Selenium, and Carotenoids. Washington, DC: National Academies Press (US). 25077263

[11] Perri G , Mendonça N , Jagger C , et al. (2020) Dietary selenium intakes and musculoskeletal function in very old adults: analysis of the Newcastle 85+ study. Nutrients 12, 1–22. 10.3390/nu12072068 PMC7400825 32664662

[12] Nève J (1995) Human selenium supplementation as assessed by changes in blood selenium concentration and glutathione peroxidase activity. J Trace Elem Med Biol 9, 65–73. 8825978 10.1016/S0946-672X(11)80013-1

[13] Xia Y , Hill KE , Li P , et al. (2010) Optimization of selenoprotein P and other plasma selenium biomarkers for the assessment of the selenium nutritional requirement: a placebo-controlled, double-blind study of selenomethionine supplementation in selenium-deficient Chinese subjects. Am J Clin Nutr 92, 525–531. 20573787 10.3945/ajcn.2010.29642 PMC2921536

[14] Hurst R , Armah CN , Dainty JR , et al. (2010) Establishing optimal selenium status: results of a randomized, double-blind, placebo-controlled trial. Am J Clin Nutr 91, 923–931. 20181815 10.3945/ajcn.2009.28169 PMC2844680

[15] Burk RF , Norsworthy BK , Hill KE , et al. (2006) Effects of chemical form of selenium on plasma biomarkers in a high-dose human supplementation trial. Cancer Epidemiol Biomarkers Prev 15, 804–810. 16614127 10.1158/1055-9965.EPI-05-0950

[16] Moschos MP (2000) Selenoprotein P. Cell Mol Life Sci 57, 1836–1845. 11215510 10.1007/PL00000665 PMC11147128

[17] Hill KE , Xia Y , Akesson B , et al. (1996) Selenoprotein P concentration in plasma is an index of selenium status in selenium-deficient and selenium-supplemented Chinese subjects. J Nutr 126, 138–145. 8558294 10.1093/jn/126.1.138

[18] Bates CJ , Thane CW , Prentice A , et al. (2002) Selenium status and its correlates in a British national diet and nutrition survey: people aged 65 years and over. J Trace Elem Med Biol 16, 1–8. 11878747 10.1016/s0946-672x(02)80002-5

[19] ter Borg S , Verlaan S , Hemsworth J , et al. (2015) Micronutrient intakes and potential inadequacies of community-dwelling older adults: a systematic review. Br J Nutr 113, 1195–1206. 25822905 10.1017/S0007114515000203 PMC4531469

[20] Akbaraly NT , Arnaud J , Hininger-Favier I , et al. (2005) Selenium and mortality in the elderly: results from the EVA study. Clin Chem 51, 2117–2123. 16123147 10.1373/clinchem.2005.055301

[21] Olivieri O , Girelli D , Azzini M , et al. (1995) Low selenium status in the elderly influences thyroid hormones. Clin Sci (London, Engl: 1979) 89, Suppl. 6, 637–642. 10.1042/cs0890637 8549083

[22] World Health Organization (1987) *Selenium: A Report of the International Programme on Chemical Safety’, Environmental Health Criteria Number 58*. Geneva: WHO.

[23] European Food Safety Authority Panel (2014) Scientific opinion on dietary reference values for selenium. EFSA J 12, 3846–3867. 10.2903/j.efsa.2014.3846 PMC13136995 42087966

[24] Collerton J , Davies K , Jagger C , et al. (2009) Health and disease in 85 year olds: baseline findings from the Newcastle 85+ cohort study. BMJ 339, b4904. 20028777 10.1136/bmj.b4904 PMC2797051

[25] Collerton J , Barrass K , Bond J , et al. (2007) The Newcastle 85+ study: biological, clinical and psychosocial factors associated with healthy ageing: study protocol. BMC Geriatr 7, 14. 17594470 10.1186/1471-2318-7-14 PMC1924857

[26] Martin-Ruiz C , Jagger C , Kingston A , et al. (2011) Assessment of a large panel of candidate biomarkers of ageing in the Newcastle 85+ study. Mech Ageing Dev 132, 496–502. 21864562 10.1016/j.mad.2011.08.001

[27] Chandola T & Jenkinson C (2000) The new UK national statistics socio-economic classification (NS-SEC); investigating social class differences in self-reported health status. J Public Health 22, 182–190. 10.1093/pubmed/22.2.182 10912557

[28] Innerd P , Catt M , Collerton J , et al. (2015) A comparison of subjective and objective measures of physical activity from the Newcastle 85+ study. Age Ageing 44, 691–694. 26018999 10.1093/ageing/afv062 PMC4476851

[29] Sales RL , Silva MM , Costa NM , et al. (2006) Development of a questionnaire to assess food intake of population groups. Rev Nutr 19, 5.

[30] Saito Y , Sato N , Hirashima M , et al. (2004) Domain structure of bi-functional selenoprotein P. Biochem J 381, 841–846. 15117283 10.1042/BJ20040328 PMC1133894

[31] Cabral M , Kuxhaus O , Eichelmann F , et al. (2021) Trace element profile and incidence of type 2 diabetes, cardiovascular disease and colorectal cancer: results from the EPIC-Potsdam cohort study. Eur J Nutr 60, 3267–3278. 33590281 10.1007/s00394-021-02494-3 PMC8354864

[32] Demircan K , Bengtsson Y , Sun Q , et al. (2021) Serum selenium, selenoprotein P and glutathione peroxidase 3 as predictors of mortality and recurrence following breast cancer diagnosis: a multicentre cohort study. Redox Biol 47, 102145. 34563873 10.1016/j.redox.2021.102145 PMC8476451

[33] Schomburg L , Orho-Melander M , Struck J , et al. (2019) Seleno protein-P deficiency predicts cardiovascular disease and death. Nutrients 11, 1852. 31404994 10.3390/nu11081852 PMC6723215

[34] Hughes DJ , Fedirko V , Jenab M , et al. (2015) Selenium status is associated with colorectal cancer risk in the European prospective investigation of cancer and nutrition cohort. Int J Cancer 136, 1149–1161. 25042282 10.1002/ijc.29071

[35] Jablan J , Besalú E , Žarak M , et al. (2021) Analytical potential of total reflection X-ray fluorescence spectrometry for simultaneous determination of iron, copper and zinc in human blood serum and plasma. Talanta 233, 122553. 34215056 10.1016/j.talanta.2021.122553

[36] Lossow K , Schlörmann W , Tuchtenhagen M , et al. (2023) Measurement of trace elements in murine liver tissue samples: comparison between ICP-MS/MS and TXRF. J Trace Elem Med Biol 78, 127167. 37004477 10.1016/j.jtemb.2023.127167

[37] Flohé L & Günzler WA (1984) Assays of glutathione peroxidase. Methods Enzymol 105, 114–121. 6727659 10.1016/s0076-6879(84)05015-1

[38] Schomburg L , Schweizer U , Holtmann B , et al. (2003) Gene disruption discloses role of selenoprotein P in selenium delivery to target tissues. Biochem J 370, 397–402. 12521380 10.1042/BJ20021853 PMC1223208

[39] Thomson CD (2004) Assessment of requirements for selenium and adequacy of selenium status: a review. Eur J Clin Nutr 58, Suppl. 3, 391–402. 14985676 10.1038/sj.ejcn.1601800

[40] Moghaddam A , Heller RA , Sun Q , et al. (2020) Selenium deficiency is associated with mortality risk from COVID-19. Nutrients 12, 2098. 32708526 10.3390/nu12072098 PMC7400921

[41] Hackler J , Demircan K , Chillon TS , et al. (2023) High throughput drug screening identifies resveratrol as suppressor of hepatic SELENOP expression. Redox Biol 59, 102592. 36586222 10.1016/j.redox.2022.102592 PMC9816962

[42] de Jong N , Gibson RS , Thomson CD , et al. (2001) Selenium and zinc status are suboptimal in a sample of older New Zealand Women in a community-based study. J Nutr 131, Suppl. 10, 2677–2684. 11584090 10.1093/jn/131.10.2677

[43] Stoffaneller R & Morse NL (2015) A review of dietary selenium intake and selenium status in Europe and the Middle East. Nutients 7, 1494–1537. 10.3390/nu7031494 PMC4377864 25734564

[44] Brown KM , Pickard K , Nicol F , et al. (2000) Effects of organic and inorganic selenium supplementation on selenoenzyme activity in blood lymphocytes, granulocytes, platelets and erythrocytes. Clin Sci 593, 593–599. 10781391

[45] Rayman MP , Thompson AJ , Bekaert B , et al. (2008) Randomized controlled trial of the effect of selenium supplementation on thyroid function in the elderly in the United Kingdom. Am J Clin Nutr 87, 370–378. 18258627 10.1093/ajcn/87.2.370

[46] Broome CS , McArdle F , Kyle JA , et al. (2004) An increase in selenium intake improves immune function and poliovirus handling in adults with marginal selenium status. Am J Clin Nutr 80, 154–162. 15213043 10.1093/ajcn/80.1.154

[47] Hargreaves MK , Liu J , Buchowski MS , et al. (2014) Plasma selenium biomarkers in low income black and white Americans from the southeastern United States. PLoS One 9, e84972. 24465457 10.1371/journal.pone.0084972 PMC3896351

[48] Department of Health (1991) *Dietary Reference Values for Food and Energy and Nutrients in the UK, Report on Health and Social Subjects’, 41*. London: HMSO. 1961974

[49] Thomson CD , Rea HM , Doesburg VM , et al. (1977) Selenium concentrations and glutathione peroxidase activities in whole blood of New Zealand residents. Br J Nutr 37, 457–460. 861195 10.1079/bjn19770049

[50] Kipp AP , Strohm D , Brigelius-Flohe R , et al. (2015) Revised reference values for selenium intake. J Trace Elem Med Biol 32, 195–199. 26302929 10.1016/j.jtemb.2015.07.005

[51] Behne D , Hilmert H , Scheid S , et al. (1988) Evidence for specific selenium target tissues and new biologically important selenoproteins. Biochim Biophys Acta 966, 12–21. 3390461 10.1016/0304-4165(88)90123-7

[52] Labunskyy VM , Hatfield DL & Gladyshev VN (2014) Selenoproteins: molecular pathways and physiological roles. Physiol Rev 94, 739–777. 24987004 10.1152/physrev.00039.2013 PMC4101630

[53] Burk RF & Hill KE (2009) Selenoprotein P-expression, functions, and roles in mammals. Biochim Biophys Acta 1790, 1441–1447. 19345254 10.1016/j.bbagen.2009.03.026 PMC2763998

[54] Schomburg L (2022) Selenoprotein P – selenium transport protein, enzyme and biomarker of selenium status. Free Radic Biol Med 191, 150–163. 36067902 10.1016/j.freeradbiomed.2022.08.022

[55] Burk RF & Hill KE (2015) Regulation of selenium metabolism and transport. Annu Rev Nutr 35, 109–134. 25974694 10.1146/annurev-nutr-071714-034250

[56] Renko K , Werner M , Renner-Müller I , et al. (2008) Hepatic selenoprotein P (SePP) expression restores selenium transport and prevents infertility and motor-incoordination in Sepp-knockout mice. Biochem J 409, 741–749. 17961124 10.1042/BJ20071172

[57] Keaney JF Jr , Larson MG , Vasan RS , et al. (2003) Obesity and systemic oxidative stress: clinical correlates of oxidative stress in the Framingham Study. Arterioscler Thromb Vasc Biol 23, 434–439. 12615693 10.1161/01.ATV.0000058402.34138.11

[58] Nichol C , Herdman J , Sattar N , et al. (1998) Changes in the concentrations of plasma selenium and selenoproteins after minor elective surgery: further evidence for a negative acute phase response? Clin Chem 44, 1764–1766. 9702974

[59] Sempértegui F , Estrella B , Vallejo W , et al. (2003) Selenium serum concentrations in malnourished Ecuadorian children: a case-control study. Int J Vitam Nutr Res 73, 181–186. 12847994 10.1024/0300-9831.73.3.181

[60] Huang Z , Rose AH & Hoffmann PR (2012) The role of selenium in inflammation and immunity: from molecular mechanisms to therapeutic opportunities. Antioxid Redox Signal 16, 705–743. 21955027 10.1089/ars.2011.4145 PMC3277928

[61] Ghayour-Mobarhan M , Taylor A , New SA , et al. (2005) Determinants of serum copper, zinc and selenium in healthy subjects. Ann Clin Biochem 42, 364–375. 16168192 10.1258/0004563054889990

[62] Gámez C , Ruiz-López D , Artacho R , et al. (1997) Serum selenium in institutionalized elderly subjects and relation to other nutritional markers. Clin Chem 43, 693–694. 9105278

[63] Monget AL , Galan P , Preziosi P , et al. (1996) Micronutrient status in elderly people. Geriatrie/Min. Vit. Aux Network. Int J Vitam Nutr Res 66, 71–76. 8698550

[64] Imai H , Suzuki T , Kashiwazaki H , et al. (1990) Dietary habit and selenium concentrations in erythrocyte and serum in a group of middle-aged and elderly Japanese. Nutr Res 10, 1205–1214.

[65] Rusip G & Suhartini SM (2020) Effects of moderate intensity exercise on glutathione peroxidase activity and VO2max in elderly women. Open Access Maced J Med Sci 8, 230–233.

[66] Powers SK & Hamilton K (1999) Antioxidants and exercise. Clin Sports Med 18, 525–536. 10410839 10.1016/s0278-5919(05)70166-6

[67] World Health Organization (2004) *Vitamin and Mineral Requirements in Human Nutrition*, 2nd ed. Geneva: World Health Organization and Food and Agriculture Organization of the United Nations. ISBN: 9241546123.

[68] Yim SH , Clish CB & Gladyshev VN (2019) Selenium deficiency is associated with pro-longevity mechanisms. Cell Rep 27, 2785–2797.e2783. 31141699 10.1016/j.celrep.2019.05.001 PMC6689410

[69] Alehagen U , Opstad TB , Alexander J , et al. (2021) Impact of selenium on biomarkers and clinical aspects related to ageing. A review. Biomolecules 11, Suppl. 10, 1478. 10.3390/biom11101478 PMC8533247 34680111

[70] Rayman MP (2012) Selenium and human health. Lancet 379, 1256–1268. 22381456 10.1016/S0140-6736(11)61452-9

[71] Lloyd B , Lloyd RS & Clayton BE (1983) Effect of smoking, alcohol, and other factors on the selenium status of a healthy population. J Epidemiol Community Health 37, 213–217. 6619720 10.1136/jech.37.3.213 PMC1052295

[72] Walsh JS , Jacques RM , Schomburg L , et al. (2021) Effect of selenium supplementation on musculoskeletal health in older women: a randomised, double-blind, placebo-controlled trial. Lancet Healthy Longev 2, e212–e221. 33842907 10.1016/S2666-7568(21)00051-9 PMC8020713

[73] Rayman MP , Stranges S , Griffin BA , et al. (2011) Effect of supplementation with high-selenium yeast on plasma lipids: a randomized trial. Ann Intern Med 154, 656–665. 21576533 10.7326/0003-4819-154-10-201105170-00005

[74] Bunker VW , Lawson MS , Stansfield MF , et al. (1988) Selenium balance studies in apparently healthy and housebound elderly people eating self-selected diets. Br J Nutr 59, 171–180. 3358921 10.1079/bjn19880024

[75] Espinoza SE , Guo H , Fedarko N , et al. (2008) Glutathione peroxidase enzyme activity in aging. J Gerontol A Biol Sci Med Sci 63, 505–509. 18511755 10.1093/gerona/63.5.505 PMC2964084

[76] Rasmussen LB , Hollenbach B , Laurberg P , et al. (2009) Serum selenium and selenoprotein P status in adult Danes - 8-year follow-up. J Trace Elem Med Biol 23, 265–271. 19747622 10.1016/j.jtemb.2009.03.009

[77] Ducros V , Faure P , Ferry M , et al. (1997) The sizes of the exchangeable pools of selenium in elderly women and their relation to institutionalization. Br J Nutr 78, 379–396. 9306881 10.1079/bjn19970158

[78] Pastori D , Pignatelli P , Farcomeni A , et al. (2016) Aging-related decline of glutathione peroxidase 3 and risk of cardiovascular events in patients with atrial fibrillation. J Am Heart Assoc 5, e003682. 27609361 10.1161/JAHA.116.003682 PMC5079030

[79] Maggio M , Ceda GP , Lauretani F , et al. (2010) Association of plasma selenium concentrations with total IGF-1 among older community-dwelling adults: the InCHIANTI study. Clin Nutr 29, 674–677. 20416996 10.1016/j.clnu.2010.03.012 PMC3963695

[80] Forte G , Deiana M , Pasella S , et al. (2014) Metals in plasma of nonagenarians and centenarians living in a key area of longevity. Exp Gerontol 60, 197–206. 25446984 10.1016/j.exger.2014.10.016

[81] Savarino L , Granchi D , Ciapetti G , et al. (2001) Serum concentrations of zinc and selenium in elderly people: results in healthy nonagenarians/centenarians. Exp Gerontol 36, 327–339. 11226746 10.1016/s0531-5565(00)00218-7

[82] Alis R , Santos-Lozano A , Sanchis-Gomar F , et al. (2016) Trace elements levels in centenarian ‘dodgers’. J Trace Elem Med Biol 35, 103–106. 27049133 10.1016/j.jtemb.2016.02.002

[83] González S , Huerta JM , Fernández S , et al. (2006) Food intake and serum selenium concentration in elderly people. Ann Nutr Metab 50, 126–131. 16391467 10.1159/000090633

[84] Brtková A , Magálová T , Babinská K , et al. (1994) Serum selenium levels in Slovak population. Biol Trace Elem Res 46, 163–171. 7888281 10.1007/BF02790076

[85] Salonen J , Alfthan G , Huttunen J , et al. (1982) Association between cardiovascular death and myocardial infarction and serum selenium in a matched-pair longitudinal study. Lancet 320, 175–179. 10.1016/s0140-6736(82)91028-5 6123886

[86] Wilhelm M , Ewers U & Schulz C (2004) Revised and new reference values for some trace elements in blood and urine for human biomonitoring in environmental medicine. Int J Hyg Environ Health 207, 69–73. 14762976 10.1078/1438-4639-00260

[87] Van Dael P & Deelstra H (1993) Selenium. Int J Vitam Nutr Res 63, 312–316. 8157441

[88] Ashton K , Hooper L , Harvey LJ , et al. (2009) Methods of assessment of selenium status in humans: a systematic review. Am J Clin Nutr 89, 2025S–2039S. 19420095 10.3945/ajcn.2009.27230F

[89] Xia Y , Hill KE & Burk RF (1989) Biochemical studies of a selenium-deficient population in China: measurement of selenium, glutathione peroxidase and other oxidant defense indices in blood. Jr Nutr 119, 1318–1326. 10.1093/jn/119.9.1318 2795246

[90] Duffield AJ , Thomson CD , Hill KE , et al. (1999) An estimation of selenium requirements for New Zealanders. Am J Clin Nutr 70, 896–903. 10539752 10.1093/ajcn/70.5.896

[91] Sunde RA , Paterson E , Evenson JK , et al. (2008) Longitudinal selenium status in healthy British adults: assessment using biochemical and molecular biomarkers. Br J Nutr 99, S37–S47. 18598587 10.1017/S0007114508006831 PMC3137460

[92] Marchaluk E , Persson-Moschos M , Thorling EB , et al. (1995) Variation in selenoprotein P concentration in serum from different European regions. Eur J Clin Nutr 49, 42–48. 7713050

[93] Persson-Moschos M , Huang W , Srikumar TS , et al. (1995) Selenoprotein P in serum as a biochemical marker of selenium status. Anal 120, 833–836. 10.1039/an9952000833 7741236

[94] Rayman MP , Taylor EW & Zhang J (2023) The relevance of selenium to viral disease with special reference to SARS-CoV-2 and COVID-19. Proc Nutr Soc 82, 1–12. 35983618 10.1017/S0029665122002646

[95] Hurst R , Collings R , Harvey LJ , et al. (2013) EURRECA-Estimating selenium requirements for deriving dietary reference values. Crit Rev Food Sci Nutr 53, 1077–1096. 23952089 10.1080/10408398.2012.742861

[96] Alfthan G , Aro A , Arvilommi H , et al. (1991) Selenium metabolism and platelet glutathione peroxidase activity in healthy Finnish men: effects of selenium yeast, selenite, and selenate. Am J Clin Nutr 53, 120–125. 1984336 10.1093/ajcn/53.1.120

[97] Giovannini S , Onder G , Lattanzio F , et al. (2018) Selenium concentrations and mortality among community-dwelling older adults: results from IlSIRENTE study. J Nutr Health Aging 22, 608–612.29717761 10.1007/s12603-018-1021-9

[98] Rayman MP , Blundell-Pound G , Pastor-Barriuso R , et al. (2012) A randomized trial of selenium supplementation and risk of type-2 diabetes, as assessed by plasma adiponectin. PloS one 7, e45269. 23028897 10.1371/journal.pone.0045269 PMC3446875

[99] Bleys J , Navas-Acien A & Guallar E (2008) Serum selenium levels and all-cause, cancer, and cardiovascular mortality among US adults. Arch Intern Med 168, 404–410. 18299496 10.1001/archinternmed.2007.74

[100] Hollenbach B , Morgenthaler NG & Struck J (2008) New assay for the measurement of selenoprotein P as a sepsis biomarker from serum. J Trace Elem Med Biol 22, 24–32. 18319137 10.1016/j.jtemb.2007.11.003

[101] Brodin O , Hackler J , Misra S , et al. (2020) Selenoprotein P as biomarker of selenium status in clinical trials with therapeutic dosages of selenite. Nutrients 12, 1067. 32290626 10.3390/nu12041067 PMC7230801

[102] Combs GF Jr , Jackson MI , Watts JC , et al. (2012) Differential responses to selenomethionine supplementation by gender and genotype in healthy adults. Br J Nutr 107, 1514–1525. 21936966 10.1017/S0007114511004715

