# Supplementary figures and images for: Selenium status and its determinants in very old adults: insights from the Newcastle 85+ Study
Source: Br J Nutr. 2023 Oct 25;131(5):901–10. doi: 10.1017/S0007114523002398 (PMC10864996; doi:10.1017/S0007114523002398)

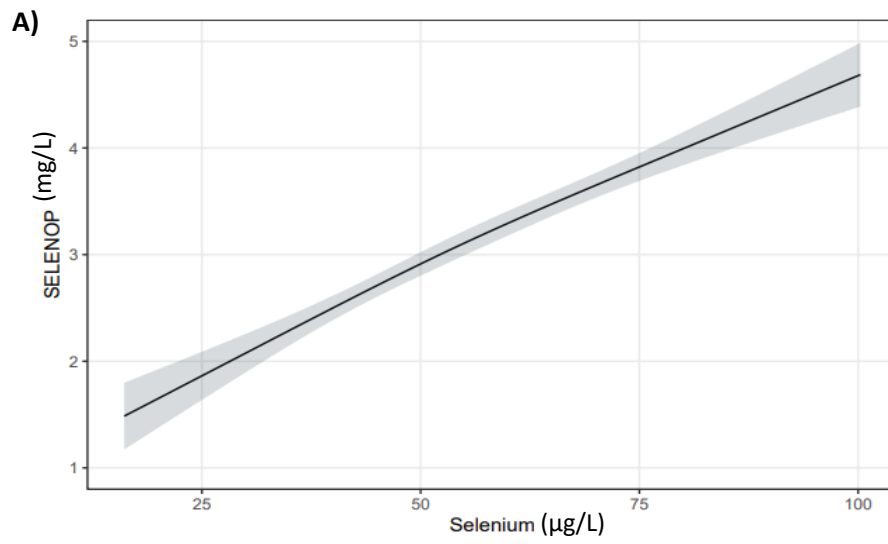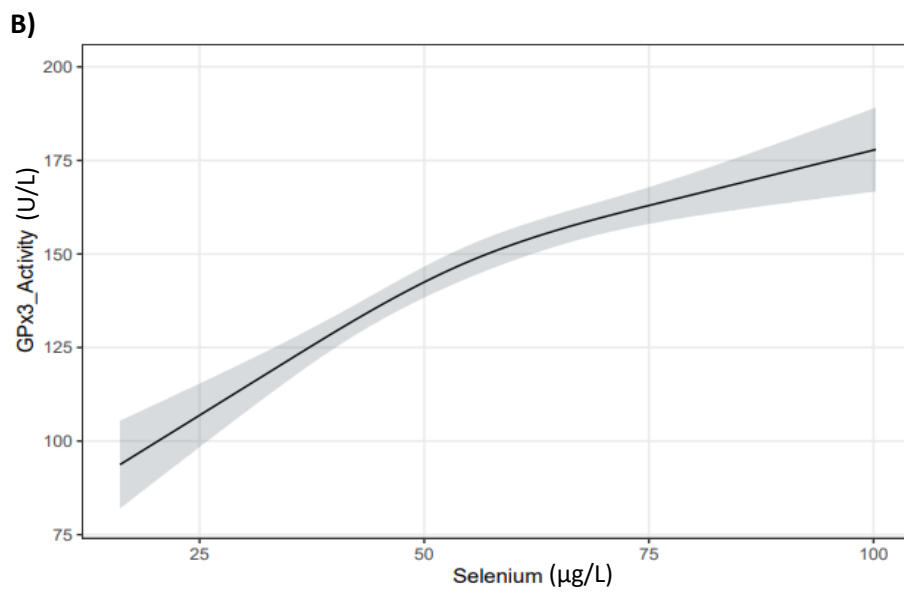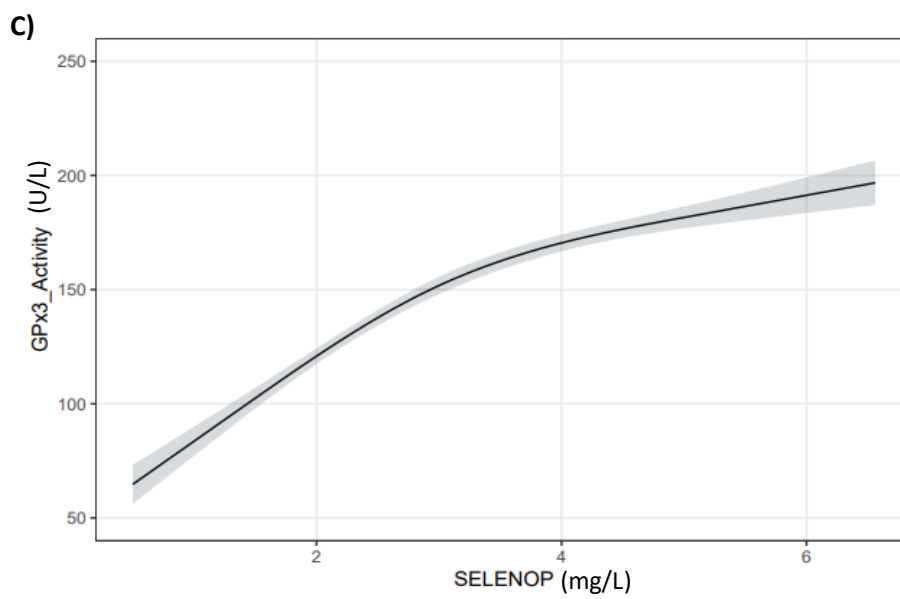

Supplement: Perri et al. supplementary material 1 — Perri et al. supplementary material [file S0007114523002398sup001.pdf]
