# Supplementary material for: Selenium status and its determinants in very old adults: insights from the Newcastle 85+ Study
Source: Br J Nutr. 2023 Oct 25;131(5):901–10. doi: 10.1017/S0007114523002398 (PMC10864996; doi:10.1017/S0007114523002398)
Supplement: Perri et al. supplementary material 2 — Perri et al. supplementary material [file S0007114523002398sup002.docx]

**Supplementary Material**

**Supplementary Table 1**: Assessments including questionnaires, measurements and blood tests taken at baseline and used in the current analyses ^(25, 26)^.

| **Assessment** | |
| --- | --- |
| Questionnaires | - Socio-economic status based on past main occupation. |
|  | - Diet |
|  | - Lifestyle (smoking, alcohol and exercise) |
| Measurements and Function Tests | - Weight |
|  | - Bio-impedance (body composition-fat and water) |
|  | - Waist and hip circumference |
|  | - Cognitive function (mini-mental state examination and computerised assessment of memory and attention (CDR battery)) |
| Blood Tests | Overnight fast to analyse: |
|  | - hsCRP |

CDR, Clinical Dementia Rating; hsCRP, high sensitivity C-reactive protein.

**Supplementary Table 2**: Diseases included in the disease count variable ^(24)^.

| **Diseases Included^a^** |
| --- |
| Hypertension |
| Ischaemic Heart Disease |
| Cerebrovascular disease |
| Peripheral vascular disease |
| Heart failure |
| Atrial flutter or fibrillation |
| Arthritis (osteoarthritis or cervical or lumbar spondylosis or rheumatoid arthritis or other arthritis or non-specified arthritis) |
| Osteoporosis |
| Chronic obstructive pulmonary disease or asthma |
| Other respiratory disease |
| Diabetes |
| Hypothyroidism or hyperthyroidism |
| Cancer diagnosed within past five years (excluding non-melanoma skin cancer) |
| Eye disease (cataract or age-related macular degeneration or glaucoma or diabetic eye disease or registered blind or partially sighted) |
| Dementia |
| Parkinson’s disease |
| Renal impairment |
| ^a^General practice diagnoses or health assessment defined ischaemic heart disease, diabetes, and thyroid disease; electrocardiogram was used to diagnose atrial fibrillation or flutter; estimated glomerular filtration rate <30 ml/min/1.73 m^2^ diagnosed renal impairment; haemoglobin concentration of < 11.5 g/dl (< 115 g/L) diagnosed anaemia. For the remaining diseases presence was derived from record review data. |

**Appendix**

| Country | Sample size | Age Range | Mean | SD | Sub-group | Mean Serum selenium concentration (µg/L) | SD or 95 % CI | Reference |
| --- | --- | --- | --- | --- | --- | --- | --- | --- |
| UK | 53 | 56-85 |  |  | Adults | 112.1 | 13.3 | ^(71)^ |
| UK | 66 | ≥ 85 |  |  | Male | 66.3 |  | ^(18)^ |
|  | 102 |  |  |  | Female | 66.3 |  |  |
| UK | 120 | 56-79 | 65.9 | 6.1 | Female | 79.4 | 15.0 | ^(72)^ |
| UK | 501 | 60-74 | 67.4 | 4.1 | Adults | 91.3 | [89.2–93.3] | ^(73)^ |
| UK | 24 | 77.7 FL |  |  | Adults | 114.5 | [109.0-120.8] | ^(74)^ |
|  | 20 | 78.8 IN |  |  | Adults | 95.5 | [85.3-106.6] |  |
| US | 571 | ≥ 65 | 77.6 | 7.8 | Female | 117.9 | 18.6 | ^(75)^ |
| Denmark | 229 | 60-65 |  |  | Male | 104.3 | 26.5 | ^(76)^ |
|  | 151 | 60-65 |  |  | Female | 98.7 | 16.8 |  |
| France | 1389 | 60-71 | 65.0 | 3.0 | Adults | 96.3 [9-year follow-up] | 7.9 | ^(20)^ |
| France | 9 | 64-82 FL | 72.0 | 2.0 | Female | 81.6 | 4.0 | ^(77)^ |
|  | 9 | 68-82 IN | 73.0 | 2.0 | Female | 65.8 | 2.2 |  |
| Italy | 749 |  | 72.8 | 8.3 | Adults | 12.0 U/ml | [6.0–21.0] | ^(78)^ |
| Italy | 951 | ≥ 65 | 75.8 | 7.5 | Adults | 74.9 | 12.6 | ^(79)^ |
| Italy | 24 | 60-63 | 61.2 | 1.1 |  | 113.9 | [103.6-124.1] | ^(80)^ |
|  | 76 | 80-99 | 89.0 | 6.3 |  | 95.0 | [90.7-99.5] |  |
|  | 64 | 100-104 | 101.0 | 1.0 |  | 84.0 | [79.5-88.7] |  |
| Italy | 62 | 60-90 | 83.4 | 2.1 | Adults | 90.0 | 17.3 | ^(81)^ |
|  | 90 | 91-110 | 94.4 | 2.4 | Adults | 68.7 | 22.1 |  |
| Italy | 46 | 70-80 |  |  | Adults | 79.2 | 26.7 | ^(82)^ |
|  | 81 | ≥ 100 |  |  | Adults | 100.0 | 38.0 |  |
| Spain | 215 |  | 75.1 | 6.5 | Adults | 86.9 | 17.4 | ^(83)^ |
|  | 88 |  | 73.4 | 7.3 | Male | 86.0 | 17.4 |  |
|  | 127 |  | 76.3 | 5.7 | Female | 87.5 | 17.4 |  |
| Europe* | 68 | ≥ 65 | 58.6 | 7.1 | Male | 75.5 | [69.7–81.8] |  |
|  | 91 |  |  |  | Female | 82.8 | [77.1-88.8] | ^(34)^ |

**Appendix Table 1:** Serum selenium concentrations of older adults in various countries.

SD, standard deviation; CI, confidence interval; FL, free living; IN, institutionalised *Denmark, France, Germany, Greece, Italy, the Netherlands, Norway, Spain, Sweden, United Kingdom

**Appendix Table 2:** Biomarkers of selenium status and reference ranges derived from the literature.

| Biomarker | Range | Reference | Source/Study | Criteria of Cut-off |  |
| --- | --- | --- | --- | --- | --- |
| Selenium  µg/L | > 45 | ^(84, 85)^ | Observational study exploring Se status in 40-60 year olds | Plasma Se cut-off of < 45 µg/L |  |
|  |  | ^(85)^ | Prospective study exploring Se status and CVD mortality in 35-59 year olds |  |  |
|  |  | ^(40)^ | Derived from healthy EPIC cohort n = 1915 | 2.5th percentile as criterion for deficient Se < 45.7 µg/L |  |
|  | 51-123 | ^(86)^ | Human Biomonitoring Commission of Germany Federal Environmental Agency | Suboptimal levels corresponded to those < 50 ng/ml (51.3 µg/L) |  |
|  | 60 | ^(87)^ | Review of 6 studies describing Se status and reference ranges | < 49.7-60.0 µg/L: low; 60-120 µg/L: normal; > 100.3-120.0 µg/L: high |  |
|  |  | ^(88)^ | ESFA DRV report: review of 18 Se supplementation studies | Studies suggsted baseline plasma Se of < 60 µg/L: low; high > 100 µg/L | |
|  | >65 | ^(39)^ | Review of assessment of requirements for Se and Se status | Plasma Se of 64.7 µg/L suggested for deiodinase activity plateau |  |
|  | 70 | ^(12)^ | Review of 7 RCT studies of selenium supplementation to explore the plateua in GPx3 activity (selenate, selenite, Se-yeast, Se-wheat, Se-meat, SeMet) | Baseline plasma Se of 60-70 µg/L was not associated with an increase in plasma GPx3 activity following supplementation suggesting plateau |  |
|  |  | ^(87)^ | Review of 2 studies exploring Se supplementation in those with higher baseline Se status | Baseline plasma Se of >69.5 µg/L suggested for GPx3 activty plateua |  |
|  |  | ^(17, 89)^ | RCT of selenate supplementation (2 wk) to explore the plateua in SELENOP in adults > 17-year-olds | Plasma Se of 70.7 µg/L suggested for SELENOP plateau |  |
|  |  | ^(6)^ | Review of country-level data for blood selenium concentrations | Used serum/plasma Se of 70 µg/L as criterion of nutritional adequacy as suggested by Ne`ve (1995) |  |
|  |  | ^(82)^ | Observational exploration of trace element comparsions between centenarians and controls in 100-104 year olds | Suggested plasma Se of 77.3 µg/L as optimal cut-off for association of being a centenarian; determined by receiver operating characteristic (ROC) curves and Youden index |  |
|  | 90 | ^(90)^ | DRV: RCT of Se supplementation (20 weeks) using SeMet (10-40 µg/d) in 19–59-year-olds adults | Plasma Se of 90 µg/L suggested for GPx3 activity plateau |  |
|  |  | ^(39)^ | Review of assessment of requirements for Se and Se status | Plasma Se of 80-95 µg/L suggested for GPx3 activity and SELENOP plateau |  |
|  |  | ^(91)^ | Longitudinal study (23 weeks) to explore serum Se and GPx3 activity correlations in 30–70-year-olds | Plasma Se of 89.0±12.6 µg/L suggested for GPx3 activity plateau |  |
|  |  | ^(92, 93)^ | Observational exploration of SELENOP maximisation using habitual diets | Plasma Se of 95-114 µg/L suggested for SELENOP plateau |  |
|  |  | ^(94)^ | Review of studies and health | Se deficiency: < 85 µg/L associated with decreased survival in HIV patients |  |
|  |  | ^(95)^ | Review of 8 studies to estimate DRV of Se | Plasma Se of 90-140 µg/L suggested for SELENOP plateau |  |
|  | 100 | ^(23)^ | DRV ESFA Report | Plasma Se of 100 µg/L suggested for GPx3 activity plateau |  |
|  |  | ^(20)^ | Prospective EVA study | Target plasma Se of 102 µg/L; Q3 and Q4 associated with improved cognition (plasma Se of 90.7-104 µg/L) |  |
|  |  | ^(39)^ | Review of Se status and optimisation of GPx3 activity and SELENOP | Plasma Se of 94.7 100 µg/L suggested for GPx3 activity plateau and CVD protection |  |
|  |  | ^(96)^ | Plotted % increments in enzyme activity in 5 supplementation studies | Plasma Se of 100-114 µg/L for GPx3 activity plateau |  |
|  |  | ^(97)^ | Observational study to explore Se status and all-cause mortality in > 80 years old | Plasma Se of < 105.3 µg/L suggested to be low Se, similar to reported concentrations for GPx3 activity plateau in Combs 1994 |  |
|  | 110 | ^(16)^ | Review of 11 studies to explore the correlations between plasma Se and SELENOP | Plasma Se of 95-118 µg/L suggested for SELENOP plateau |  |
|  |  | ^(14)^ | RCT of Se yeast or Se-enriched onions (12 weeks) in 50–64-year-olds | Plasma Se of 125 µg/L suggested for SELENOP plateau |  |
|  | 120/118 | ^(6)^ | Review of Se status for cancer protection  Requirement derived using an equation from a RCT of Se supplementation (Yang et al. 1989) | Plasma Se of 120 µg/L associated with cancer prevention  Y = 1.623logX+3.433  Y = daily selenium intake; X = plasma selenium |  |
|  |  | ^(39)^ | Review of assessment of requirements for Se and Se status | Plasma Se of > 118.4 µg/L for protection against some cancers |  |
|  | >120 | ^(98)^ | Review of Se and cancer from 4 studies | Plasma Se of > 122 µg/L provides no further cancer protection |  |
|  |  | ^(70)^ | Review of Se and health using data from NHANES ^(99)^ | Plasma Se of 130-150 µg/L associated with minimal mortality |  |
| SELENOP mg/L | >2.56 | ^(40)^ | Derived from healthy EPIC cohort n = 1915 | 2.5th percentile as criterion for deficient SELENOP < 2.56 mg/L |  |
|  | 3.0 | ^(100)^ | Obsrvational study to explore the use of SELENOP as sepsis biomarker | Standards with plasma Se of 98.4 µg/L had SELENOP of 4.4 mg/L, median values of healthy adults 3.0 mg/L |  |
|  | 3.2 | ^(76)^ | Observational study of Se status in 60-65 year olds | Baseline SELENOP in 60-65 year old adults with serum Se approx 100 µg/L |  |
|  | 3.3 | ^(17)^ | RCT of Se supplementation using standards for analyses | Estimated SELENOP in reference plasma |  |
|  | 3.7-4.6 [4.3] | ^(34)^ | Prospective EPIC Europe ≥ 65 years | Geometric mean ranges of healthy adult controls; males: 3.9 [3.7-4.2]; females: 4.3 [4.0-4.5]; total 20-70 years: 4.3 [2.9-6.1] |  |
|  | 4.3 | ^(33)^ | Prospective Malmö Preventive Project 70 years | Quintile 1 associated with increased risk of CVD and mortality |  |
|  | 4.7 | ^(47)^ | Observational study of Se status in US 40-79 year olds | Mean concentration of 4.7 mg/L with baseline plasma Se of 115 µg/L |  |
|  | 5.5 | ^(15)^ | RCT Se supplementation (sodium selenite, Se-yeast and L-SeMet) moderate to high doses (200-600 µg/d) (16 week) | Derived from analysis of the human standard plasma in selenium-replete subjects with baseline plasma Se of 122 µg/L |  |
|  | 5.0-7.0 | ^(13, 14, 101, 102)^ | RCTs of 3 Se supplementation studies up to 400 µg/d for maximal SELENOP expression | Suggested maximal concentrations for SELENOP plateau |  |
|  | 6.35 | ^(14)^ | RCT of Se yeast or Se-enriched onions (12 weeks) in 50–64-year-olds | Suggested concentrations at SELENOP plateau |  |
|  | >10 | ^(101)^ | Review of SECAR and SOS-LVAD RCTs of intravenous selenite (> 1 mg/d) | Suggested maximal concentrations for SELENOP plateau |  |
| GPx3 Activity  U/L | 115 | ^(32)^ | SCAN-B cohort: breast cancer survivors, mean GPx3 activty of 209±47 U/L | Determined the 2.5^th^ centile to detect low values |  |
|  | 123-195 | ^(86)^ | HBM 2002 | Reference values of 123-167 U/L f females; 127-195 U/L for males |  |
|  | 136 | ^(47)^ | Observational study of Se status in US 40-79 year olds | Mean concentration of 136 U/L with baseline plasma Se of 115 µg/L |  |
|  | 159 | ^(15)^ | RCT Se supplementation (sodium selenite, Se-yeast and L-SeMet) moderate to high doses (200-600 µg/d) (16 wk) | Baseline GPx3 activity of 159 U/L suggested as reference |  |

Se, selenium; CVD, cardiovascular disease; HBM, Human Biomonitoring; ESFA, European Food Safety Authority; DRV, dietary recommended value; RCT, randomised controlled trial; GPx3, glutathione peroxidase activity; SeMet, selenomethionine; SELENOP, selenoprotein P.

**Appendix Table 3:** The characteristics of study participants represented by deficient selenium status cut-offs.

| Characteristic | | All Participants  n = 757 | | Selenium  < 45 µg/L | | | | Selenium ≥ 45 µg/L | | *p* | SELENOP  < 2.6 mg/L | | | SELENOP  ≥ 2.6mg/L | | *p* |
| --- | --- | --- | --- | --- | --- | --- | --- | --- | --- | --- | --- | --- | --- | --- | --- | --- |
| *Socio-demographic factors* |  | | | |  |  |  |  |  |  |  |  |  |  |  |  |
| Women % (n) | | 61.1 | 461 | 31.0 | | 143 | | 69.0 | 318 | 0.78 | 39.0 | 180 | | 61.0 | 281 | 0.60 |
| Men % (n) | | 38.9 | 293 | 30.0 | | 88 | | 70.0 | 205 |  | 41.0 | 120 | | 59.0 | 173 |  |
| *Education % (n)* n = 743 | | | | | | | | | | | | |  |  |  |  |
| *0-9 years* | | 64.2 | 477 | 31.9 | | 152 | | 68.1 | 325 | 0.25 | 36.9 | 176 | | 63.1 | 301 | 0.04 |
| *10-11 years* | | 23.6 | 175 | 30.9 | | 54 | | 69.1 | 121 |  | 48.0 | 84 | | 52.0 | 91 |  |
| *12+ years* | | 12.2 | 91 | 23.1 | | 21 | | 76.9 | 70 |  | 40.7 | 37 | | 59.3 | 54 |  |
| *Living in Institution* % (n) n = 755 | | | | | | | | | | | | |  |  |  |  |
| Yes | | 8.9 | 67 | 68.7 | | 46 | | 31.3 | 21 | <0.001 | 49.3 | 33 | | 50.7 | 34 | 0.10 |
| No | | 91.1 | 688 | 27.0 | | 186 | | 73.0 | 502 |  | 39.0 | 268 | | 61.0 | 420 |  |
| *Diet-related factors* | | | | | | | | | | | | |  |  |  |  |
| Total energy kCal (M, SD) n = 732 | | 1688.6 | 511.0 | 1695.5 | | 506.1 | | 1704.5 | 508.5 | 0.46 | 1698.7 | 505.7 | | 1704.2 | 509.2 | 0.23 |
| Protein intake g (M, SD) n = 732 | | 64.2 | 22.3 | 62.0 | | 20.0 | | 66.0 | 23.1 | 0.03 | 62.8 | 21.7 | | 66.2 | 22.6 | 0.005 |
| Selenium Intake µg/d (M, SD) n = 732 | | 45.3 | 29.8 | 42.8 | | 36.8 | | 47.6 | 28.7 | <0.001 | 45.3 | 28.5 | | 46.9 | 32.9 | 0.16 |
| *Lifestyle factors* | | | | | | | | | | | | |  |  |  |  |
| Number of Medications (M, SD) n = 732 | | 6.3 | 3.8 | 7.1 | | 3.5 | | 5.7 | 3.5 | <0.001 | 6.1 | 3.6 | | 6.1 | 3.6 | 0.84 |
| *Total Medication* n % n = 753 | | | | | | |  | | | | | | | | | |
| 0-2 | | 16.7 | 126 | 20.6 | | 26 | | 79.4 | 100 | <0.001 | 43.7 | 55 | | 56.3 | 71 | 0.40 |
| 3-5 | | 26.7 | 201 | 24.4 | | 49 | | 75.6 | 152 |  | 36.3 | 73 | | 63.7 | 128 |  |
| ≥6 | | 56.6 | 426 | 36.6 | | 156 | | 63.4 | 270 |  | 40.4 | 172 | | 59.6 | 254 |  |
| Alcohol drinker % (n) n = 751 | | | | | | | | | | | | |  |  |  |  |
| Yes | | 62.3 | 468 | 27.1 | | 104 | | 72.9 | 179 | 0.006 | 41.5 | 105 | | 58.5 | 178 | 0.24 |
| No | | 37.7 | 283 | 36.7 | | 127 | | 63.3 | 341 |  | 37.1 | 194 | | 62.9 | 274 |  |
| Physical activity score % (n) n = 748 | | | | | | | | | | | | |  |  |  |  |
| Low | | 21.7 | 162 | 48.1 | | 78 | | 51.9 | 84 | <0.001 | 44.4 | 72 | | 55.6 | 90 | 0.07 |
| Medium | | 43.0 | 322 | 26.1 | | 84 | | 73.9 | 238 |  | 41.9 | 135 | | 58.1 | 187 |  |
| High | | 35.3 | 264 | 25.4 | | 67 | | 69.4 | 197 |  | 34.5 | 91 | | 65.5 | 173 |  |
| *Health-related factors* | | | | | | | | | | | | |  |  |  |  |
| *Self-rated health* n = 738 | | | | | | | | | | | | |  |  |  |  |
| Excellent/Very Good | | 40.9 | 302 | 25.5 | | 77 | | 74.5 | 225 | 0.07 | 37.7 | 114 | | 62.3 | 188 | 0.29 |
| Good | | 37.7 | 278 | 32.7 | | 91 | | 67.3 | 187 |  | 43.2 | 120 | | 56.8 | 158 |  |
| Fair/Poor | | 21.4 | 158 | 34.2 | | 54 | | 65.8 | 104 |  | 36.7 | 58 | | 63.3 | 100 |  |
| SMMSE (M, SD) n = 753 | | 26.1 | 4.9 | 26.1 | | 4.6 | | 27.1 | 3.4 | <0.001 | 26.8 | 4.0 | | 26.8 | 3.7 | 0.91 |
| hsCRP mg/L (M, SD) n = 753 | | 6.9 | 14.2 | 7.2 | | 15.7 | | 5.6 | 12.8 | 0.005 | 6.6 | 15.9 | | 5.7 | 12.0 | 0.08 |
| Free T_4_ pmol/L (M, SD) n = 742 | | 15.6 | 2.7 | 15.9 | | 2.7 | | 15.5 | 2.6 | 0.16 | 16.1 | 2.6 | | 15.3 | 2.6 | <0.001 |
| Free T_3_ pmol/L (M, SD) n - 743 | | 4.5 | 0.5 | 4.4 | | 0.5 | | 4.6 | 0.5 | <0.001 | 4.6 | 0.6 | | 4.5 | 0.5 | 0.74 |
| *Anthropometry* | | | | | | | | | | | | |  |  |  |  |
| BMI (M, SD) n = 674 | | 24.4 | 4.4 | 24.3 | | 4.4 | | 24.5 | 4.3 | 0.59 | 24.7 | 4.3 | | 24.2 | 4.3 | 0.29 |
| Fat Free Mass (M, SD) n = 689 | | 45.2 | 9.0 | 18.3 | | 7.7 | | 19.1 | 7.7 | 0.96 | 19.3 | 7.5 | | 18.6 | 7.8 | 0.62 |
| Waist:Hip Ratio (M, SD) n = 685 | | 0.89 | 0.08 | 0.88 | | 0.07 | | 0.88 | 0.08 | 0.68 | 0.89 | 0.07 | | 0.88 | 0.07 | 0.13 |

The characteristics are displayed for all participants and those with concentrations above and below cut-offs for deficient/non-deficient selenium and selenoprotein status. SELENOP, selenoprotein P; M, mean; SD, standard deviation; SMMSE, standardised mini mental state examination; hsCRP, high sensitivity C-Reactive protein; Free T4, free thyroxine; Free T3, free triiodothyronine; BMI, body mass index. P values determined using Mann Whitney U or Kruskal Wallis tests for continuous variables or Chi -square test for categorical variables. P values indicate the difference between deficient and non-deficient status.
